# DNA-free RNA preparations from mycobacteria

**DOI:** 10.1186/1471-2180-4-45

**Published:** 2004-11-30

**Authors:** Joachim Stephan, Johannes G Bail, Fritz Titgemeyer, Michael Niederweis

**Affiliations:** 1 Lehrstuhl für Mikrobiologie, Friedrich-Alexander-Universität Erlangen-Nürnberg, Staudtstr. 5, D-91058 Erlangen, Germany; 2 Department of Microbiology, University of Alabama at Birmingham, 609 Bevill Biomedical Research Building, 845 19th Street South, Birmingham, AL 35294, USA

## Abstract

**Background:**

To understand mycobacterial pathogenesis analysis of gene expression by quantification of RNA levels becomes increasingly important. However, current preparation methods yield mycobacterial RNA that is contaminated with chromosomal DNA.

**Results:**

After sonication of RNA samples from *Mycobacterium smegmatis *genomic DNA is efficiently removed by DNaseI in contrast to untreated samples.

**Conclusions:**

This procedure eliminates one of the most prevalent error sources in quantification of RNA levels in mycobacteria.

## Background

*Mycobacterium tuberculosis *is the leading cause of death from a single infectious disease with approximately 8.8 million new cases and two million deaths per year. To understand the pathogenesis of *M. tuberculosis*, analysis of gene expression by relative or absolute quantification of RNA levels using microarrays and RT-PCR (batch- and real-time) becomes increasingly important [1]. Widely used methods to isolate bacterial RNA are acid-phenol extraction or guanidinium isothiocyanate extraction combined with cesium chloride purification or nucleic acid binding resins [2]. However, the cell wall of mycobacteria is very stable and a very effective permeability barrier, and, therefore, rather refractory to lysis by chaotropic agents and detergents, hampering RNA isolation from these microorganisms [3]. Since the average half-life of mycobacterial mRNA is in the range of a few minutes, mycobacteria have to be vigorously treated (e.g. bead-beating, freeze-thawing, nitrogen decompression) to quickly isolate RNA [4]. This causes fragmentation of chromosomal DNA that contaminates RNA preparations, which is one of the most prevalent error sources in quantification of RNA levels in mycobacteria. Several methods have been suggested to circumvent this problem [5,6]. Virtually all RNA isolation protocols use DNaseI, which does not completely remove large amounts of DNA. Our goal was to improve the efficiency of DNaseI digestion by solubilizing chromosomal DNA with sonication prior to DNaseI treatment. *Mycobacterium smegmatis *is especially refractory to lysis and therefore was chosen as a model organism.

## Results and Methods

**Figure 1:**
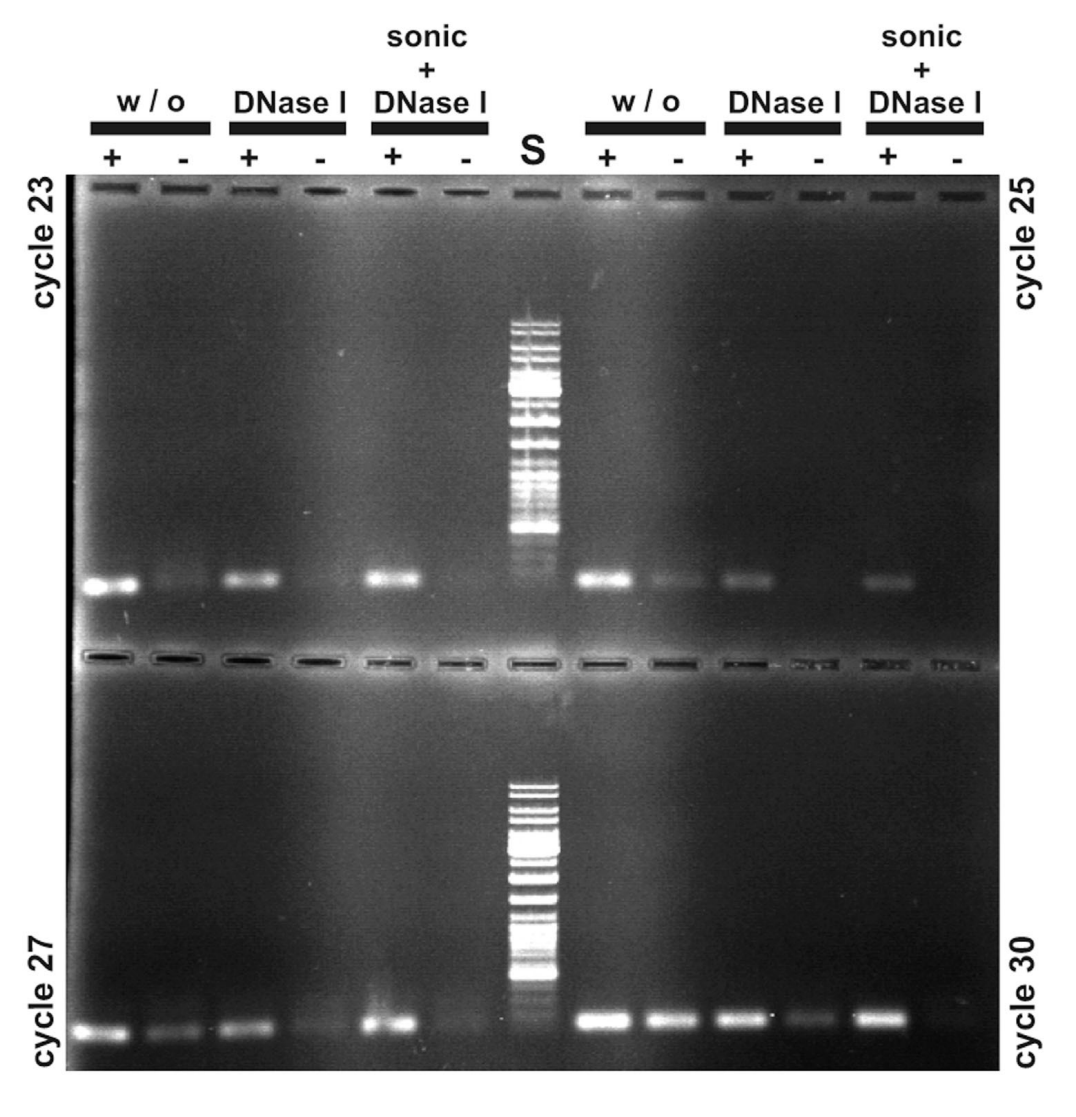
**Analysis of DNA contaminations in a RNA preparation from *M. smegmatis *by RT-PCR **. One-tenth of the volume after RT-PCR was loaded on a 1% agarose gel, which was stained with ethidium bromide. Three different samples for RT-PCR were used: an untreated RNA (indicated as w/o), a DNaseI treated preparation (DNaseI) and a sonicated and DNaseI treated extraction (sonic + DNaseI). The four gels show samples taken after cycle 23, 25, 27 and 30 of the PCR. For every RT-PCR a negative control (-) was performed, in which the RNA was added to the sample after the RT step.

*M. smegmatis *SMR5 [7] was grown in 10 ml Middlebrook 7H9 liquid medium (Difco Laboratories; supplemented with 0.2% glycerol, 0.05% Tween 80) to an OD_600 _of 0.8 and mixed with 5 ml killing buffer (20 mM Tris-HCl, 5 mM MgCl_2_, 20 mM NaN_3_) [8]. The cell suspension was incubated on ice for 5 min. Cells were harvested by centrifugation (20 min at 6000 × g and 4°C). 20 mg cells (dry weight) were lysed in FastRNA Blue-Tubes (Bio-101 Inc.) using a FastPrep FP120 bead-beater apparatus (Savant, USA) for 20 sec at level 6.5. The tubes were centrifuged for 10 min at 10000 × g and 4°C. The supernatant was transferred to microcentrifuge tubes containing a nucleic acid binding resin (Nucleospin RNA II; Macherey-Nagel), and further experimental steps were done as described by the manufacturer. A total of 62 μg RNA was eluted in 60 μl of RNase-free water. The RNA was diluted to 50 ng μl^-1 ^into several aliquots. One aliquot containing 10 μg RNA was left untreated. The second aliquot was directly treated with 10U of RNase-free DNaseI (Roche) for 1 h at 37°C, while the third aliquot was sonicated two times for 20 sec with 0.9 sec intervals at 20 % power (Sonopuls HD 2070; Bandelin electronic) prior to DNaseI treatment. Between the two sonication steps the cell suspension was chilled on ice for 5 min. DNaseI was removed by precipitation with polyethylene glycol (PEG) 6000. As a control for the RNA quality, cDNA was synthesized by Omniscript reverse transcriptase and sensiscript reverse transcriptase (OneStep RT-PCR system, QIAGEN) from total RNA (100 ng) for 35 min at 50°C followed by an inactivation step of 15 min at 95°C. The 16Sr RNA was then amplified with the primers 16S-FP (5'-TGCTACAATGGCCGGTACAAA-3') and 16S-RP (5'-GCGATTACTAGCGACGCCGACTT-3') using up to 30 cycles of 1 min at 94°C, 30 sec at 53°C, and 1 min at 72°C before a final extension step of 7 min at 72°C. As a control for DNA contamination, standard PCRs were performed, to which RNA was added after the RT inactivation step. PCR products were analysed at cycles 23, 25, 27 or 30 to check the purity of the RNA. All samples apparently contained 16S rRNA (Fig. 1). However, DNA contamination was detected by PCR at cycle 25 in the RNA sample that was not treated with DNaseI. Conventional DNaseI treatment delayed the appearance of a signal in the sample without the RT step until cycle 27. By contrast, no amplification product was obtained in the control sample even after 30 cycles, when the RNA sample was sonicated before DNaseI treatment (Fig. 1).

## Conclusions

This result shows that sonication improved DNA degradation by DNaseI most likely by rendering the chromosomal DNA more accessible to enzymatic action. This work describes a simple and efficient procedure to improve the quality of RNA preparations from *M. smegmatis *and will be of great value for RNA preparations from other microorganisms, including *M. tuberculosis*.

## Authors' contributions

JS carried out the RNA preparations, the RT-PCR experiments and wrote a draft of the manuscript. JGB performed RNA preparations and RT-PCR experiments. FT participated in coordinating and supervising the study. MN conceived of the study, participated in coordinating and supervising the study, and wrote the final manuscript. All authors read and approved the final manuscript.
